# Toward safer and sustainable food preservation: a comprehensive review of bacteriocins in the food industry

**DOI:** 10.1042/BSR20241594

**Published:** 2025-04-17

**Authors:** José Carlos Parada Fabián, Ana Karen Álvarez Contreras, Iván Natividad Bonifacio, Marcos Francisco Hernández Robles, Carlos Ramón Vázquez Quiñones, Elsa Irma Quiñones Ramírez, Carlos Vázquez Salinas

**Affiliations:** 1 Departamento de Microbiología, Escuela Nacional de Ciencias Biológicas-IPN, Santo Tomás, Manuel Carpio, Plutarco Elías Calles, Miguel Hidalgo, C.P. 11350 CDMX, Mexico city, Mexico; 2 Departamento de Biotecnología, Universidad Autónoma Metropolitana – Iztapalapa, San Rafael Atlixco 186, Leyes de Reforma 1A Sección, Iztapalapa, C.P. 09310 CDMX, Mexico City, Mexico

**Keywords:** antimicrobial activity, bacteriocins, biopreservatives, control of pathogens, food preservation

## Abstract

Bacteriocins are considered promising natural biopreservatives in the food industry because of their broad spectrum of antimicrobial activity against Gram-positive bacteria and foodborne pathogens. This review provides information on several bacteriocins (nisin, pediocin, Micocin^®^, lacticin 3147, and enterocin AS-48), their mechanisms of action, applications, and discussion of regulatory requirements for their approval as food additives by the Food and Drug Administration (FDA) and the European Union to improve food safety. Nisin (the most studied bacteriocin), recognized as generally regarded as safe by the FDA, is used as a food preservative. Pediocin, derived from *Pediococcus acidilactici*, shows efficacy against *Listeria* species and is used in vegetable and meat products. Micocin^®^, a mixture of bacteriocins produced by *Carnobacterium maltaromaticum* CB1, is effective against *Clostridium botulinum* and *Listeria monocytogenes*. Lacticin 3147, composed of two peptides: Ltnα and Ltnβ, shows synergistic antibacterial activity with potential applications in the control of pathogens in dairy products. Enterococcin AS-48, produced by *Enterococcus faecalis* subsp. *liquefaciens* S-48, exhibits broad-spectrum antimicrobial activity against several Gram-positive bacteria and has been studied for biopreservation in a number of food products. For regulatory approval, the following criteria must be met: determination of identity, chemical composition, safety assessments, and recommended concentrations for use. Despite the difficulties posed by their large-scale production and purification, bacteriocins hold enormous potential for improving food safety and shelf life; however, further research is required to harness bacteriocins as future food preservation strategies

## Introduction

Microbial activity is a critical factor in food spoilage, as it can degrade the quality, safety, and shelf life of food products [1]. A wide range of microorganisms, including bacteria, molds, and yeasts, contribute to spoilage by producing enzymes or metabolic by-products that alter food’s sensory and nutritional properties [2]. Common spoilage microbes include *Pseudomonas* sp., *Lactobacillus* sp., *Clostridium* sp., *Penicillium* sp., and *Saccharomyces* sp., which thrive under different environmental conditions and substrates, making food preservation a complex challenge [3].

In 2023, the World Health Organization (WHO) published global estimates on foodborne diseases (FBDs), highlighting that more than 200 of these diseases are caused by bacteria, viruses, parasites, or chemical substances [4,5]. Each year, these conditions result in approximately 600 million cases and around 420,000 deaths worldwide [6,7]. In addition to their impact on health, FBDss cause significant economic losses, estimated at 110 billion dollars due to reduced productivity and healthcare costs [8]. The globalization of food trade demands efficient communication between governmental authorities when unsafe products enter international markets. To address this challenge, the FAO and WHO established the International Network of Food Safety Authorities (INFOSAN) in 2004. This network, comprising more than 600 members from 188 member states, facilitates rapid information exchange and contributes to strengthening food safety systems, thereby reducing the global burden of FBDs [9].

The collaboration and complementarity between global networks like INFOSAN and PulseNet play a crucial role in enhancing the efficiency and impact of worldwide initiatives to reduce FBDs. Today, food safety and quality have become critical priorities, driving the development of preservation methods that not only maintain freshness and flavor but also address the challenges posed by globalization and evolving consumer demands [10]. Over recent decades, lactic acid bacteria (LAB) have gained attention as natural biopreservatives in dairy products, thanks to their ability to produce a variety of metabolites, including lactic acid, hydrogen peroxide, diacetyl, and bacteriocins, the latter being of particular interest [11].

Bacteriocins produced by LAB have garnered significant interest in the food industry due to their generally recognized as safe (GRAS) status, specifically in the case of the nisin [12]. Their ability to inhibit foodborne pathogens and spoilage microorganisms, combined with their safety for human consumption, positions them as a promising alternative to conventional antimicrobials [13]. These ribosomally synthesized antimicrobial peptides exhibit either lytic or inhibitory activity against microorganisms. To become active, they often require post-translational modifications, including structural changes involving amino acids such as lanthionine and methyl lanthionine or unsaturated amino acids, which enable the formation of intramolecular ring structures, typically through disulfide bonds [14,15]. It is worth mentioning that not all ribosomally synthesized peptides are considered bacteriocins, as these peptides can perform various other functions [16]. Bacteriocins are effective against a broad spectrum of bacterial species through multiple mechanisms of action, further enhancing their potential as antimicrobial agents [17]. The rising demand for bacteriocins stems from their suitability as food preservation additives; however, their large-scale application is hindered by challenges such as low production yields and difficulties in purification during fermentation processes [18,19]. There are three primary approaches to using bacteriocins for food biopreservation: inoculating foods with bacteriocin-producing strains, incorporating purified or semi-purified bacteriocins as food additives, or utilizing fermented products containing bacteriocin-producing strains as ingredients in food processing [20]. Currently, the U.S. Food and Drug Administration (FDA) has approved bacteriocins such as nisin, pediocin, and Micocin® for use as food additives, though other bacteriocins also hold great potential for future applications in food preservation [17]. In summary, bacteriocins represent a crucial tool for enhancing food safety and shelf life. Promoting their broader application requires addressing the challenges associated with their large-scale production and purification while emphasizing their GRAS status, efficacy, and diverse applications in food biopreservation.

## Bacteriocin-producing microorganisms

Most bacteriocins are primarily produced by Gram-positive bacteria; however, Gram-negative bacteria also synthesize antimicrobial peptides with comparable properties [21]. Research indicates that most bacterial species can produce at least one type of bacteriocin, although a significant number of these molecules remain unidentified [22]. In the case of enterobacteria, the bacteriocins they produce are commonly referred to as colicins [15]. The discovery of the first bacteriocin dates to 1925, when it was identified in *Escherichia coli* [23]. Colicins are present in 30–50% of *E. coli* strains isolated from human hosts and have since become a model system for studying various aspects of bacteriocins, including their structure, function, genetic organization, and evolutionary history [22,24].

Among the bacteriocins, those produced by enterobacteria, such as colicins, and by LAB have been the subject of extensive research. This focus has led to a wealth of scientific studies, especially regarding their potential industrial applications. Bacteriocins from the LAB group have garnered significant interest due to their promising use in food preservation and safety [25,26].

Bacteriocins exhibit a high degree of specificity due to their distinct amino acid sequences, which provide precise complementarity with the surface proteins of target pathogens. This molecular recognition allows bacteriocins to bind selectively to specific receptors on the bacterial cell membrane, leading to the disruption of essential cellular processes. As a result, their mode of action is highly targeted, minimizing effects on non-susceptible bacteria and making them promising candidates for antimicrobial applications [27].

### Lactic acid bacteria

LAB are Gram-positive microorganisms distinguished by their ability to produce lactic acid as the primary product of fermentation [28]. Morphologically, they appear as cocci or bacilli, are non-spore-forming, catalase-negative, and function either as facultative anaerobes or microaerophiles [29]. These heterotrophic bacteria exhibit high nutritional demands for amino acids and vitamins, as evolutionary reductions in their biosynthetic capabilities restrict their growth to nutrient-rich environments [30,31].

Facultatively heterofermentative LAB, such as *Leuconostoc* species and specific *Lactobacillus* strains, play a crucial role in traditional food industries, particularly in processes like cheese ripening. These bacteria metabolize hexoses via the Embden–Meyerhof–Parnas (EMP) pathway, producing lactic acid and fermenting other sugars to yield volatile organic acids, which contribute to the flavor and preservation of fermented foods [28,32,33]. LAB are naturally found in a wide variety of habitats, including decomposing plant matter, dairy products, fermented meats and fish, cereals, pickled vegetables, silages, fermented beverages, juices, wastewater, and the oral and gastrointestinal cavities of humans and animals [34].

Taxonomically, LAB are classified within the phylum *Firmicutes*, class *Bacilli*, and order *Lactobacillales* [35]. Their classification is based on characteristics such as cell morphology, pathways of glucose fermentation, optimal growth temperatures, and sugar utilization patterns. Prominent genera include *Lactobacillus*, *Lactococcus*, *Leuconostoc*, *Pediococcus*, *Streptococcus*, and others, with *Lactobacillus* being the most diverse, encompassing over 100 species commonly found in carbohydrate-rich environments [36].

### Fermentative characteristics

The metabolism of LAB integrates both fermentation and respiration through a mechanism known as extracellular electron transfer. During respiration, ATP synthesis occurs either through oxidative phosphorylation or through substrate-level phosphorylation, while maintaining a redox balance. Homofermentative LAB are capable of mixed-acid fermentation, whereas heterofermentative LAB rely on alternative electron acceptors, such as citrate, for their metabolic processes [35].

LAB can be classified into two main groups based on their fermentation end products: homofermentative and heterofermentative. The heterofermentative group is further divided into facultative and strict fermentative species [37]. Strictly homofermentative LAB, including certain species of *Pediococcus*, *Lactococcus*, *Streptococcus*, and *Lactobacillus*, exclusively ferment hexoses, converting them almost entirely into lactic acid via the EMP pathway. However, not all these species are capable of degrading pentoses [38].

In contrast, strictly heterofermentative LAB lack the glycolytic enzyme fructose-1,6-bisphosphate aldolase, which prevents them from metabolizing hexoses through the EMP pathway. Instead, they utilize the phosphogluconate pathway to degrade hexoses, producing not only lactic acid but also significant quantities of ethanol or acetic acid, along with carbon dioxide, as fermentation by-products [39].

### Methods for isolation and identification of LAB

LAB have significant nutritional requirements, including carbohydrates, amino acids, and various growth factors [40]. Their cultivation often relies on protein-rich media, as LAB growth is closely linked to the availability of nitrogen sources. An optimal carbon-to-nitrogen ratio of approximately 5:1 is essential, with proteins and yeast extract serving as primary nitrogen sources to support biomass production and the synthesis of secondary metabolites [41].

Several specialized culture media are commonly used for LAB isolation, including Man Rogosa Sharpe (MRS) agar, which is ideal for general LAB cultivation, Rogosa Agar (LBS) for *Lactobacillus* spp., M17 Agar for *Lactococcus* spp., and MSE medium for *Leuconostoc* spp. By selecting the appropriate medium and adjusting incubation conditions, targeted isolation of specific LAB strains can be achieved [42].

MRS agar is particularly recommended for isolating LAB due to its unique composition. The inclusion of citrate and sodium acetate supports biomass production by entering the Krebs cycle, while Tween 80 influences the fatty acid composition of bacterial membranes. Additionally, manganese salts provide essential cofactors for enzymatic activity [43,44]. On MRS agar, LAB typically form small, pinpoint white colonies, although some colonies may reach 2–3 mm in diameter [45]. Alternative culture media enriched with waste-derived compounds, such as cabbage liquor, have also shown promise for LAB cultivation. These sustainable approaches provide viable options for biomass production while reducing environmental impact [41].

### Phenotypic identification

The phenotypic identification of LAB isolates can be conducted through conventional biochemical tests or micromethods, which may be semi-automated or fully automated [46]. Traditional biochemical identification relies on key characteristics, including microscopic morphology, catalase and oxidase activity, carbohydrate fermentation profiles, tolerance to varying sodium chloride concentrations (1.5–10%), growth at different pH levels (3.0–8.5) and temperatures (15–45°C), arginine hydrolysis, citrate utilization, and lactic acid production. However, this approach involves significant costs due to the number and complexity of tests required [28]. To assess fermentative capabilities, phenol red broth supplemented with various carbohydrates is commonly used [47].

Micromethods offer a more efficient alternative for LAB identification by reducing the material and time required, making them more cost-effective than conventional techniques. Additionally, they allow for the inclusion of a greater number of biochemical tests, which can be repeated for accuracy [42]. A notable example is the standardized gallery system, specifically designed to study carbohydrate metabolism for the identification of *Lactobacillus* and related genera [38]. These methods provide a reliable and economical approach for LAB characterization while maintaining high accuracy.

### Genotypic identification

The *Lactobacillus* genus is taxonomically complex, comprising over 170 species that are phenotypically indistinguishable and require molecular methods for detection. This diversity makes *Lactobacillus* a highly heterogeneous group, and as such, the most reliable and preferred method for identifying species within this genus is the use of molecular techniques, particularly the identification of genes such as 16S rRNA [48].

Several molecular techniques have been developed to identify LAB. These include DNA-DNA hybridization, plasmid analysis, restriction fragment length polymorphism analysis, and pulsed-field gel electrophoresis of genomic DNA. Additionally, rRNA gene sequencing, particularly of the 16S rRNA gene, plays a crucial role in establishing phylogenetic relationships among species. Other methods include PCR and techniques based on probe usage, which can be specific for *in situ* hybridization or universal approaches such as ribotyping [49]. These molecular techniques provide accurate and detailed identification of LAB, overcoming the limitations of traditional phenotypic methods.

### Antimicrobial components produced by LAB

LAB produce a wide range of antimicrobial compounds, including organic acids (such as lactic acid, citric acid, acetic acid, fumaric acid, and malic acid), hydrogen peroxide, diacetyl, ethanol, bacteriocins, and other significant metabolites (Table 1). These substances exhibit potent antagonistic activity against various microorganisms. Additionally, the antimicrobial properties of LAB are partly due to their competition with pathogenic microorganisms for available nutrients [4].

**Table 1:** Mechanisms of action of various antimicrobial compounds produced by lactic acid bacteria.

Antimicrobial compounds	Mechanisms of action	References
Organic acids	Reduce intracellular pH, disrupting cellular functions and affecting enzymatic activity. Additionally, they can dissociate and cause damage to cell membranes.	[50]
Hydrogen peroxide	Generates highly reactive free radicals, leading to oxidative damage to cellular biomolecules, resulting in the disruption of essential cellular functions.	[51]
Diacetyl	Interferes with protein and nucleic acid synthesis, affecting cellular function and DNA replication.	[52]
Ethanol	Causes protein denaturation and damage to cell membranes, disrupting cellular homeostasis and functionality of cellular components.	[53]

The production of lactic acid and other organic acids leads to a decrease in the pH of the surrounding environment, which inhibits the growth of both Gram-positive and Gram-negative bacteria. Lactic acid, in its undissociated form, can easily penetrate the microbial cell wall. Once inside, the higher pH within the cell causes the acid to dissociate, releasing hydrogen ions and the corresponding anion. These ions interfere with the cell’s metabolic processes, thereby inhibiting growth and contributing to the antimicrobial effects of LAB [54].

Furthermore, when oxygen is present, LAB can produce hydrogen peroxide, which generates hydroxyl radicals that cause peroxidation of membrane lipids, leading to increased susceptibility of the bacterial cell [55].

## Bacteriocins

Bacteriocins are a diverse family of ribosomally synthesized peptides with a strong antimicrobial activity at specific concentrations. These peptides are typically produced as biologically inactive precursor molecules containing an N-terminal leader sequence. In some cases, they undergo post-translational modifications before the leader sequence is cleaved, allowing the active peptide to be exported from the cell [56,57].

To date, several hundred bacteriocins have been identified, with evidence suggesting that 99% of all bacteria can produce them, often more than one type, many of which remain undiscovered [58]. Bacteriocins generally exhibit a bacteriostatic or bactericidal effect on bacteria closely related to the producing strain; though in some cases, they can target a broader range of unrelated bacterial species. To avoid self-harm, bacteriocin-producing bacteria have developed protective mechanisms such as immunity proteins, efflux pumps, or combinations of both [59,60].

**Figure 1:**
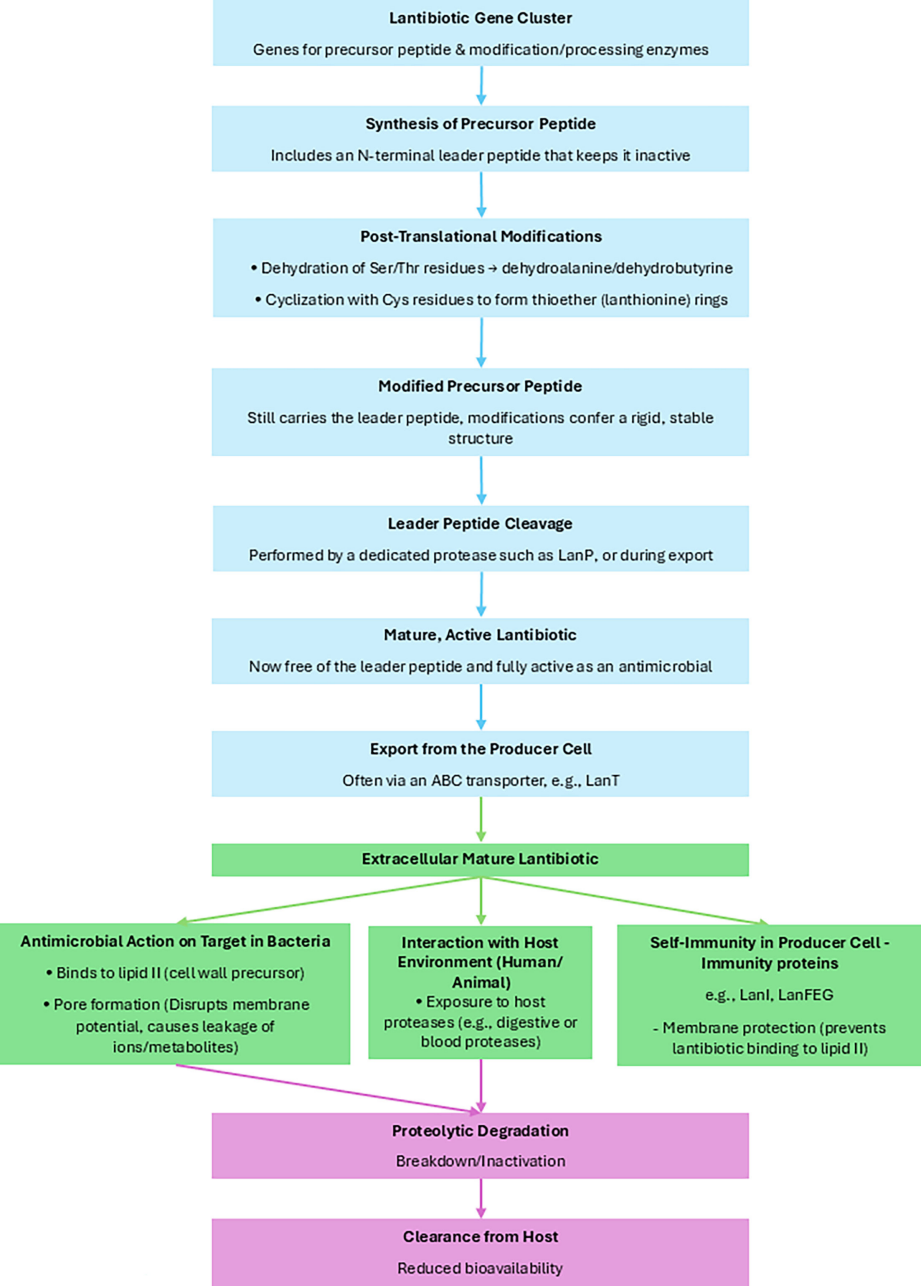
Schematic representation of lantibiotic biosynthesis, maturation, antimicrobial action, and self-immunity mechanisms in the producer cell. The biosynthetic process begins with gene cluster transcription and precursor peptide synthesis, which includes an N-terminal leader peptide preventing premature activation. Post-translational modifications, such as dehydration and lanthionine bridge formation, enhance structural stability. Leader peptide cleavage by a specific protease (e.g., LanP) activates the lantibiotic, which is then exported via an ABC transporter (e.g., LanT). The mature lantibiotic exerts its antimicrobial effect by binding to lipid II, disrupting cell wall synthesis, and/or forming membrane pores. In a host (human/animal), proteolytic degradation by digestive or systemic proteases reduces its bioavailability and contributes to its clearance. To avoid self-toxicity, bacteriocin-producing bacteria co-express immunity proteins (e.g., LanI and/or LanFEG system), which prevent the lantibiotic from binding to lipid II on the producer’s cell membrane. These immunity proteins may function by modifying lipid II, sequestering inadvertently produced lantibiotics, or acting as efflux pumps, ensuring that the bacteriocin remains active against competing, non-immune bacteria while protecting the producer cell.

Bacteriocins produced by LAB have been extensively studied for their antimicrobial activity against pathogens such as *Listeria monocytogenes*, *Staphylococcus aureus*, *Bacillus cereus*, *C. botulinum*, and *Salmonella* sp., among others (Figure 1) [22].

Bacteriocins are characterized by the following properties:

Safety: They are proteinaceous and digested in the gastrointestinal tract by proteolytic enzymes like trypsin, α-chymotrypsin, and pepsin. Some bacteriocins remain stable in gastric conditions but degrade in the small intestine, while others are sensitive to stomach environments and deactivate during digestion [61].

Potency: Effective at nanomolar or picomolar concentrations, especially against microorganisms closely related to the producing bacteria [62].

Heat resistance: Stable under typical heat treatments used in pasteurization or sterilization [63].

Despite their potential and high efficacy, the broader application of bacteriocins is limited by challenges such as high production costs, low yields, and the complex technological requirements for their synthesis [64].

### Bacteriocin production

Bacteriocins are classified into three classes: class 1, lantibiotics; class II, non-lantibiotics; and class III, heat-stable and large-sized bacteriocins. This classification is based on the biochemical and genetic characteristics of these molecules [65].

A bacterial strain produces a type of bacteriocin that is associated with the growth conditions; for example, the *Lactococcus lactis* subsp. *cremoris* 9 B4 produces Lactococcin B (non-lantibiotics) and its production is influenced by environmental factors, the availability of nutrients, the presence of acetic acid, signaling peptides, as well as the cell density reached in the logarithmic phase of growth [62]. The genes responsible for bacteriocin synthesis are found on chromosomes, plasmids, or transposons. These peptides are initially ribosomally synthesized in a biologically inactive form and later undergo modifications to become active [66]. Typically, the genes encoding bacteriocin production and immunity are organized in operon clusters and are often associated with mobile genetic elements such as transposons or plasmids. Newly synthesized bacteriocins include an N-terminal leader peptide [36].

Bacteriocins are ribosomally synthesized, biologically inactive peptides composed of an N-terminal leader peptide attached to a C-terminal pre-peptide. After synthesis, they are post-translationally modified. The genes involved in the transport and maturation of pre-peptides are located near those responsible for bacteriocin biosynthesis [22,67]. Leader peptide plays a critical role as a recognition site, directing the pre-peptide to maturation and transport proteins. It also protects the producing strain by keeping the bacteriocin inactive within the cell and interacts with the pre-peptide domain to ensure proper conformation for the enzyme–substrate interactions required for its modification [68].

### Classification

Since Klaenhammer’s foundational classification of bacteriocins in 1993 [69], several updated systems have been proposed. One of the most recent classifications, introduced by Álvarez-Sieiro et al. in 2016 [54], categorizes bacteriocins based on their biosynthetic mechanisms, genetic features, and structural characteristics. This system divides bacteriocins into three main classes: class I and class II, which are heat-stable peptides smaller than 10 kDa, and class III, which consists of thermolabile peptides larger than 10 kDa (Table 2).

**Table 2:** Classes and properties of bacteriocins from lactic acid bacteria.

Class	Average size (kDa)	Main amino acids	Principal characteristics	Microorganism involve	Application	Reference
I	<5	Lanthionine, methyl-lanthionine, dehydroalanine, dehydrobutyrine	Small, thermostable, with unusual amino acids. They can be linear or globular.	*Lactococcus lactis*, *Streptomyces* sp.	Food preservation, antimicrobial agents in the pharmaceutical industry.	[70]
IIa	<10	TGNGVXC (consensus)	Active against *Listeria monocytogenes*	*Pediococcus acidilactici*	Control of *L. monocytogenes* in foods.	[71]
IIb	<10		They form complexes to act.	*Lactiplantibacillus plantarum*	Preservation of fermented vegetables.	[63]
IIc	<10		Small, thermostable, transport by peptide.	*Lactococcus lactis*	Preservation of dairy products.	[72]
III	>30		Large, thermolabile, complex structure.	*Lactobacillus helveticus*	Biocontrol agents in agriculture.	[73]

### Class I: lantibiotics

Class I bacteriocins, known as lantibiotics, are antimicrobial peptides with molecular weights of less than 5 kDa. These peptides are thermally stable, resistant to extreme pH levels, and immune to certain proteases [70]. The name ‘lantibiotics’ derives from ‘lanthionine,’ a unique amino acid formed when two alanine residues are linked by a thioether bond [74,75]. A key characteristic of lantibiotics is their post-translationally modified amino acids, such as lanthionine, methyl-lanthionine, dehydroalanine, and dehydrobutyrine. These modifications contribute to the rigidity, resistance to proteolytic degradation, and thermal stability of the peptides [68,76].

Lantibiotics are further classified into three structural and functional types: linear (type A), globular (type B), and multicomponent (type C) [68]. Their biosynthesis is encoded by gene clusters that include structural genes for pre-lantibiotic peptides, as well as genes responsible for modification, export, regulation, and immunity [70].

One of the most well-known lantibiotics is nisin, produced by *Lactococcus lactis*. It was the first bacteriocin to be purified and requires 11 genes for its synthesis [54,77]. The nisABTCIP operons (biosynthesis and immunity) and nisFEG operons (immunity) are regulated by the NisRK two-component system, which acts as a quorum-sensing mechanism responding to nisin concentrations [54].

Nisin biosynthesis begins with the translation of the *nisA* gene into pre-nisin A. This precursor is then modified by the products of the *nisB* and *nisC* genes to form the nisin A precursor [78]. Nisin consists of 34 amino acids and contains five rings formed by disulfide bridges [79]. The precursor is exported with the assistance of the *nisT* and *nisP* genes, where the leader peptide is cleaved to activate nisin A [80].

Bacteriocin expression can be regulated by external induction factors, which are often secreted by the producing strain, or it may occur constitutively. The biosynthesis of bacteriocins is influenced by environmental conditions such as temperature and pH [81]. Non-lantibiotic bacteriocins differ slightly in their biosynthesis, as they do not require the incorporation of unusual amino acids [54].

### Mechanism of action

Bacteriocins primarily target the bacterial cytoplasmic membrane, where they act on energized membrane vesicles to disrupt the proton motive force [82]. Lantibiotics have a cationic nature, which enables them to interact with the plasma membrane of the target cell. This interaction leads to the formation of pores in the membrane, reducing the proton motive force [68]. The process begins with the electrostatic attraction of bacteriocins to the target bacteria. Since bacteriocins are positively charged, they interact with the negatively charged phospholipids of the bacterial membrane [82]. Additionally, the amphipathic nature of bacteriocins enhances their distribution across the surface of the bacterial cell membrane [83].

### Binding of bacteriocins to target bacteria

Bacteriocins bind to target bacteria by interacting between their hydrophilic N-terminal region and the polar surface of the bacterial cell membrane. Once bound, the hydrophobic C-terminal region of the bacteriocin penetrates the non-polar interior of the membrane. This interaction causes pore formation in the membrane, leading to the loss of potassium ions (K+), ATP energy, and, in some cases, amino acids and small molecules [84].

Certain class I bacteriocins, like nisin, exhibit a dual mode of action [85]. They can form pores in the membrane, where the N-terminal beta-sheet domain binds to the cell membrane, while the C-terminal region penetrates the membrane, causing leakage. In addition to pore formation, nisin can bind to lipid II, which is essential for the transport of peptidoglycan subunits, thereby inhibiting cell wall synthesis [68,86].

### Lantibiotic maturation

Lantibiotics undergo a series of maturation steps, involving conformational changes mediated by several enzymes acting on the ribosomally synthesized pre-peptide [80]. In the first stage of maturation, the dehydratase NisB modifies the nisin precursor by dehydrating serine and threonine residues through glutamylation and tRNA-dependent elimination [87]. The cyclase NisC facilitates the formation of methyllanthionine rings via a Michael-type nucleophilic attack from a cysteine thiol group onto a dehydrated residue at the N-terminal end. The modified precursor is then transported and proteolytically activated by the NisT and NisP enzymes. In some Type I lantibiotics, this maturation process may occur intracellularly or through an unidentified leader peptidase not associated with the lantibiotic group [54].

### Class II: non-lantibiotics or unmodified peptides

Class II bacteriocins are small (<10 kDa), cationic, hydrophobic, heat-stable peptides that do not undergo post-translational modifications. These bacteriocins are divided into three subclasses: IIa, IIb, and IIc [68,88].

### Class IIa: pediocin-like bacteriocins

Class IIa bacteriocins, also known as pediocin-like bacteriocins, are the most well-known antimicrobial peptides produced by LAB. They are listericidal, small (<10 kDa), heat-stable, and unmodified, consisting of 37–48 amino acids [17]. The genes responsible for their production are typically organized in operon clusters, including a structural gene for the pre-peptide, an immunity gene, an ABC transporter gene for secretion, and sometimes an accessory protein gene. Occasionally, regulatory genes are also present [36].

The most studied Class IIa bacteriocin is pediocin PA-1. Its gene cluster is plasmid-encoded and contains four genes: *pedA* (structural gene), *pedB* (immunity gene), and *pedC* and *pedD* (ABC transporter and accessory protein, respectively). The operon produces two transcripts: a smaller one for pedABC and a larger one for pedABCD. The leader peptide functions as a recognition signal for processing and secretion by the ABC transporter [54].

### Class IIb: two-peptide bacteriocins

Class IIb bacteriocins require the presence of two distinct peptides for full antimicrobial activity, with both peptides needed in roughly equal amounts. The production of these bacteriocins involves at least five genes, organized into one or two operons [63].

An example of a Class IIb bacteriocin is lactococcin G, whose operon contains two structural genes for pre-bacteriocins, an immunity gene, a gene for a dedicated ABC transporter, and another gene for an accessory protein with an unclear function. The structural genes are adjacent and co-transcribed, ensuring that both peptides are produced in equal amounts. These peptides exhibit minimal antimicrobial activity when acting individually but work synergistically to exert a potent antimicrobial effect when combined. Their production is regulated by a three-component quorum sensing (QS) system, involving an induction factor, a membrane-associated histidine kinase, and response regulators [89,90].

### Class IIc: leaderless bacteriocins

Class IIc bacteriocins are unique in that they lack the N-terminal leader peptide that is typically present in other bacteriocins. The leader peptide functions as a recognition sequence for secretion and modification, maintaining the bacteriocin in an inactive state within the producing cell [34].

### Mechanism of action

The production of bacteriocins generally involves multiple genes responsible for various functions such as amino acid modification, export, regulation, and immunity. These genetic systems can be located on bacterial chromosomes, plasmids, or conjugative transposons [91]. Typically, bacteriocin-producing bacteria synthesize autoimmunity proteins that protect them from being harmed by their own bacteriocins [76].

### Mechanism of action of class II bacteriocins

The mechanism of action of class II bacteriocins varies depending on the specific bacteriocin, but generally, they disrupt the cytoplasmic membrane of target bacteria through pore formation or degradation of the cell wall [68]. Some bacteriocins, like pediocin (class IIa), have been shown to use the mannose phosphotransferase permease systems (Man-PTS) as receptors. These systems are present in both Gram-positive and Gram-negative bacteria and serve as binding sites for various antibacterial macromolecules, including bacteriocins [92]. The binding of pediocin to the MptC and MptD subunits of the Man-PTS facilitates the insertion of the bacteriocin into the target cell’s membrane. This results in the irreversible opening of an intrinsic channel, allowing ion diffusion across the membrane, ultimately leading to cell death [85].

### Class IIb bacteriocins: two-peptide systems

Class IIb bacteriocins, consisting of two distinct unmodified peptides, cause membrane permeabilization and pore formation in sensitive bacteria. These pores are selective for monovalent cations, such as Na^+^, K^+^, Li^+^, Cs^+^, and Rb^+^, as seen in lactococcin G [83].

### Class IIc bacteriocins: circular peptides

Class IIc bacteriocins are circular peptides with a positive net charge. These bacteriocins interact directly with the negatively charged bacterial membrane, eliminating the need for receptor molecules. This interaction leads to pore formation, ion efflux, and dissipation of the membrane potential, ultimately causing cell death [85].

### Class III bacteriocins

Class III bacteriocins are large peptides (>30 kDa) that can be either heat-labile and lytic or non-lytic in nature. Some examples include zoocin A, lysostaphin, and helveticin J and V [73]. The antibacterial activity of class III bacteriocins is primarily due to their enzymatic functions, such as endopeptidase activity, which targets and disrupts the bacterial cell wall. Class III bacteriocins are further divided into two subclasses: IIIa (bacteriolysins) and IIIb [67].

### Subclass IIIa: bacteriolysins

Subclass IIIa includes bacteriocins that lyse bacterial cells by degrading the cell wall. A well-known example of this subclass is lysostaphin, produced by *Staphylococcus simulans* biovar *staphylolyticus* [93,94]. These bacteriocins act by cleaving the peptidoglycan in the bacterial cell wall, leading to cell lysis.

### Subclass IIIb: non-lytic peptides

Subclass IIIb bacteriocins do not cause cell lysis but instead disrupt the plasma membrane potential of the target bacteria [20]. These bacteriocins interfere with membrane integrity, which may result in membrane depolarization and loss of cellular function, but without causing direct breakdown of the cell wall.

## Immunity mechanisms

Bacteriocin-producing strains protect themselves from the harmful effects of their own bacteriocins by producing specific immunity proteins. These immunity proteins are often encoded by genes that are closely linked to the bacteriocin production genes [95]. In many cases, the immunity and structural bacteriocin genes are located within the same operon conformed generally by two or three genes [96]. Some of them encoded to transcriptional regulator and molecules involved in transporting. For example, the Lan I and Lan EFG systems, a multicomponent ABC transporter, have been described for Lan I. This protein helps protect the producer cells by preventing pore formation. It achieves this by pushing the bacteriocin molecules out of the membrane and maintaining a controlled concentration of the bacteriocin, thus ensuring that the producer cell is not harmed by its own antimicrobial peptides [36]. *Lactococcus lactis* and *Bacillus subtilis* produce and transport nisin and subtilin, respectively, from the membrane to the extracellular space; and *Bacillus thuringiensis* carries the *thnR* gene that encodes a transcriptional regulator [97].

## Mechanisms of resistance to bacteriocins

Prolonged or frequent exposure to bacteriocins produces resistant strains due to selection pressure, although there are also bacteria that, without being previously exposed to bacteriocins, present a resistance naturally. According to Bastos et al. [98], resistance to bacteriocins is classified as innate or acquired. The mechanisms of innate resistance are electrostatic charge of the cell wall and alterations in the structure of the membrane, while the mechanisms of acquired resistance are genetic mutations, alterations in the expression of receptors, and production of degradative enzymes. All reports on resistance mechanisms have been made using nisin as a study model.

### Innate resistance

#### Modification of the electrostatic charge of the cell wall

The negative net charge of the cell wall of bacterial cells is given by the presence of teichoic and lipoteichoic acids. However, some Gram-positive microorganisms can esterify the phosphate groups of teichoic and lipoteichoic acid with D-alanine, producing a change in the negative net charge of the cell wall. The presence of the *dlt* operon is responsible for the modification of the charge of the cell wall, and the *dltA* gene has a main role as transporter of the amino acid to the cytoplasm and as a ligase to join it to teichoic and lipoteichoic acid. The modification of the negative charge in the cell wall of Gram-positive microorganisms causes a lower electrostatic interaction with bacteriocins, which have a positive net charge. This resistance mechanism has been studied in *S. aureus, Clostridium difficile,* and *B. cereus* in the presence of nisin [99–101].

### Alteration in membrane composition

The alteration of the membrane is an innate mechanism of some bacteria to decrease the action of nisin. This has been observed in strains of *L. monocytogenes* by synthesizing more phosphatidylglycerol, while decreasing the synthesis of diphosphatidylglycerol, causing changes in the charge and fluidity of the membrane. Nisin, having a net positive charge, can insert itself into the membrane with a net negative charge and form pores. However, when there is an alteration in the phosphatidylglycerol/diphosphatidylglycerol ratio and other membrane lipids, the net negative charge is altered, in addition to becoming more rigid in regions of curvature of the cell (microdomain), making it difficult for nisin to insert into those areas where it normally has greater affinity [102].

### Acquired resistance

#### Genetic mutations

In *L. monocytogenes* strains, it has been observed that the mutation in the LiaFSR signaling pathway increases resistance to the action of nisin because it is related to the alteration of the membrane and metabolism in the presence of environmental factors that generate stress in the microorganism. *L. monocytogenes* in the presence of nisin enters a state of stress, activating the LiaFSR pathway which is a cell-enveloped stress-sensing three-component system that activates the genes related to structural changes in the membrane and cell wall [103].

### Alteration of receptor expression

It has been observed that the LiaFSR system in *L. monocytogenes* strains is a group of genes involved in the adaptive response to antimicrobial agents such as nisin. Within this system, the *LiaH* and *LiaI* genes have a protective role in the membrane, stabilizing the structure of the cell through anchoring proteins, while the *LiaR* gene alters the synthesis of type II phospholipid in the membrane, considered a nisin receptor [104].

### Production of degradative enzymes

*B. cereus* and *B. polymyxa* are some of the microorganisms capable of producing nisinases during the sporulation phase. This enzyme can break the C-terminal of the lanthionine ring, inactivating nisin because it cannot bind to the membrane receptor [105].

### Purification and identification of bacteriocins

Various extraction and purification strategies for bacteriocins have been developed, with methods tailored to their specific nature, similar to other proteins [79]. It is noteworthy that these antibacterial activity peptides are characterized by being produced during the logarithmic growth phase, and their highest presence is observed during the late logarithmic phase [83].

### Purification

The most important and complex stage is purification. Currently, the choice of purification method is determined by the molecular weight, charge, and properties of the bacteriocin [14]. Some purification methods used in different studies include the following:

- Ammonium sulfate precipitation: used to precipitate bacteriocins from an aqueous solution by adding ammonium sulfate. Bacteriocins are recovered as a precipitate that can be further purified [106].
- Ion exchange chromatography (IEC): used to separate charged bacteriocins from other molecules with charge in a sample, based on the electrostatic interaction between molecules and charged resins [66].
- Size exclusion chromatography: used to separate bacteriocins according to their molecular size. It relies on the ability of larger molecules to elute before smaller molecules [107].
- Affinity chromatography: used to separate bacteriocins that specifically bind to a target molecule. It relies on the ability of molecules to bind to a specific affinity matrix [108].

Most bacteriocins detected in recent years have been obtained through precipitation with ammonium sulfate, using IEC or RP-HPLC [72,109]. However, since there is no universal method, the process depends entirely on the characteristics of the bacteriocin of interest [14,68].

The first step required for bacteriocin purification starts with a step to concentrate bacteriocins from the culture supernatant, using, for example, diatomite calcium silicate or ammonium sulfate precipitation [110]. The first step required for bacteriocin purification involves concentrating on the supernatant, assuming an optimized bacteriocin production process. Some of them are found in molecular aggregates; these macromolecules are disaggregated using agents that dissociate the macromolecules, ultrafiltration, or by removing lipid material through extraction with methanol–chloroform or ethanol–diethyl ether [82]. Bacteriocins from the supernatant can be concentrated according to their size: filtration, precipitation with ammonium sulfate, and extraction with organic solvents such as butanol and ethanol [83].

### Identification

The search for new compounds involves the use of many strains; the efficiency and accuracy of the analysis are the most important factors. One of the methods that meets these criteria is PCR analysis, which allows for the rapid and easy identification of genes encoding bacteriocin [111]. The bacteriocin PCR matrix is based on enterococcal genes related to known bacteriocin from the NCBI GenBank database. This method is currently used to detect bacteriocin-producing bacteria. However, it is important to note that different producer strains may have different genetic sequences, requiring the selection of specific primer pairs for each of them [14]. Identifying bacteriocin-producing strains uses physiological and biochemical criteria, as well as 16S rRNA gene analysis. It is also important to study the supernatant at different pH values, temperatures, and enzymatic resistance [112].

### Bacteriocin genes and detection

Genes involved in bacteriocin biosynthesis are organized in clusters within the bacterial genome and include those encoding pre-bacteriocins, enzymes for post-translational modification, immunity proteins, and transport systems for export [113].

Generally, the gene that generates the signal precursor and genes encoding the membrane sensor protein and the response regulator responsible for detecting it are found in the same operon [114]. The peptide precursor is processed, and the resulting signal peptide (AIP) is exported to the cell exterior through a transporter protein, which is usually of the ABC type [115]. In some cases, the AIP is modified during excretion. The peptide signal is generally detected outside the cell by a membrane sensor kinase that transmits the signal by phosphorylation to a response regulator that will act directly on the transcription of target genes and QS system genes, initiating a cycle of auto-induction. In some cases, such as in *Enterococcus faecalis* or *B. subtilis*, the AIP is internalized through a specific transporter protein after being modified and secreted [116].

Detection of these proteins or their genes provides an indication of the biochemical machinery responsible for bacteriocin biosynthesis and can be detected using molecular techniques [117]. Additionally, genomic sequencing and bioinformatics analysis facilitate the identification and characterization of new bacteriocin genes, contributing to the development of new antimicrobial additives [118].

### Bacteriocin databases

Currently, there are various query databases for the topic of bacteriocins, where different types of analyses can be performed for these molecules, such as phylogenetic analysis, molecule identification, structure prediction, and genomic-level evaluation of the presence of genes encoding this type of molecule [20].

### BAGEL4 database

BAGEL4 is a web server for the identification and visualization of gene clusters in prokaryotic DNA related to the biosynthesis of ribosomally synthesized and post-translationally modified peptides (RiPPs) and unmodified bacteriocins. This tool is useful for the challenging task of identifying genes responsible for bacteriocin and RiPP production [119].

The bacteriocin database contains nearly 500 RiPPs (class I), 230 unmodified bacteriocins (class II), and 90 large bacteriocins (>10 kDa) (class III). Most records contain a link to NCBI or UniProt; BAGEL4 requires a nucleotide sequence in FASTA format as input, allowing multiple files and entries per file, with an upload limit of 50 Mb [120]. It also offers the option to select a genome from a list containing complete genomes (WGS), using the name or RefSeq accession number. The sequence is analyzed only if its read length exceeds the established minimum (default 3000 bp) [121].

The process begins with the translation of DNA into six large proteins, starting from a valid start codon (ATG, GTG, or TTG); since various types of non-AUG start codons have been identified, it is essential to consider them when determining ORFs (Open Reading Frames) [122]. The resulting proteins are analyzed for specific protein motifs and compared with the central peptide database, identifying areas of interest (AOIs) [119]. Once AOIs are identified, they are analyzed in detail to detect ORFs using Gene Locator and Interpolated Markov ModelER (Glimmer3), a tool specifically designed for gene identification in microbial DNA, including bacterial, archaeal, and viral genomes. Glimmer3 employs interpolated Markov models to locate coding regions and distinguish them from noncoding DNA. It also identifies and labels small ORFs located within intergenic regions [123]. These ORFs are compared with the annotation database and the central peptide database, producing alignments, and predicting promoters and terminators. General results include links to detailed reports per AOI, containing graphical visualizations of annotated genes, promoters, and terminators. An alignment is also shown if homology with a record in the central peptide database is found, along with structural data from UniProt if available [119].

### LABiocin

LABiocin is a database specialized in bacteriocins from LABs, where important data are compiled, including name, class, amino acids, and nucleic acid sequences involved in bacteriocin synthesis, if available [124]. The LABiocin platform provides comprehensive data on antimicrobial peptides, including details about target microorganisms, source organisms, strain status, and specific cultivation conditions. Additionally, it offers information on the methods used for peptide extraction and purification, enabling researchers to replicate or optimize procedures [125]. This detailed metadata supports comparative studies on antimicrobial activity across different strains and experimental conditions, facilitating advancements in biotechnological applications and the development of novel antimicrobial agents. Additionally, a phylogenetic tree is available for mature peptide bacteriocin sequences [126].

In addition to data search tools, it features a BLAST tool in the database to allow the user to perform a homology search with mature peptide sequences. Users can link to other databases containing additional information, particularly on predicted bacteriocin structure and mechanisms of action [124].

This tool has served as a foundation and inspiration for developing specialized databases such as abAMPdb, a resource dedicated to identifying antimicrobial peptides in *Acinetobacter baumannii* [127]. It has also supported the detection of various bacteriocins in *E. faecium* and *E. lactis* [128], as well as expression assays in LAB cultures [129].

### Application of bacteriocins

Indigenous LABs have been reported as an efficient alternative for natural biopreservation and the prevention of FBDs [130]. Over the past two decades, research on LAB strains, their antibacterial products (bacteriocins), and their potential applications in food biopreservation has significantly increased, bringing about new changes in the use of natural products for food preservation [131].

Modern consumers are more aware than previous generations of the negative health effects associated with the consumption of certain chemical preservatives and the significant effects of heat on the nutritional value and taste of many foods [132].

As ready-to-eat and minimally processed foods become staples of the modern diet, the food industry faces an unprecedented challenge to provide foods that are not only convenient but also safe, nutritious, and free from harmful microorganisms [133,134]. The increasing demand for these foods comes with heightened consumer expectations for extended shelf life, natural ingredients, and minimal preservatives [135]. Ensuring food safety in this context requires innovative preservation techniques and rigorous quality control to prevent contamination and spoilage while maintaining sensory and nutritional quality [136]. This challenge is critical for public health and for meeting regulatory standards, prompting ongoing research into antimicrobial agents and preservation methods that align with consumer preferences for healthier, ‘clean-label’ products [137,138].

As a result, the food industry is under pressure to employ innovative processing methods to meet consumer regulatory demands [61]. As natural antimicrobial agents, bacteriocins present an appealing alternative to chemical preservatives, especially in response to the growing consumer demand for safe, minimally processed, and ready-to-eat foods [139]. Bacteriocins offer targeted antimicrobial activity against harmful bacteria, enhancing food safety without the need for synthetic additives [50,140]. Since bacteriocins are colorless, odorless, and tasteless, they can be incorporated into food products without altering their organoleptic properties [10].

### Food biopreservation

Biopreservation is a sustainable approach to improving food safety and maintaining or extending the life of foods using beneficial microorganisms or their metabolites [8]. Food biopreservation encompasses a range of techniques aimed at producing safer foods while enabling the creation of minimally processed, additive-free products [51,130]. These methods utilize natural antimicrobial agents, such as bacteriocins, organic acids, and protective cultures, to inhibit spoilage organisms and pathogens, enhancing food safety without compromising quality [52]. Biopreservation aligns with consumer demand for clean-label and naturally preserved foods, offering an effective approach to extend shelf life while avoiding synthetic additives [53]. The implementation of modern technologies in food processing and microbiological safety assurance has decreased but has not eliminated the risks of diseases related to the consumption of foods contaminated with microorganisms [141].

Currently, there are many control measures within the food industry to prevent or minimize contamination by bacteria, including good manufacturing practices, effective sanitation and hygiene measures regarding raw materials, and other hazard analysis and critical control points (HACCP) fundamentals [142]. These measures allow for the identification, assessment, and control of significant hazards to food safety [143]. However, despite these precautions, FBDs occur with alarming frequency. For example, *L. monocytogenes* is a significant pathogen for the food industry and susceptible consumers, including pregnant women, infants, immunosuppressed individuals, and adults over 65 years of age. *L. monocytogenes* is ubiquitous in the environment and extremely resistant, surviving refrigeration temperatures and high salt concentrations [12].

This underscores the substantial risk that this pathogen presents to both consumers and the food industry [144]. Implementing additional protective measures in food products to prevent such outbreaks is crucial [145]. Bacteriocins offer an economically viable solution, serving as natural antimicrobial agents that can enhance food safety by effectively inhibiting pathogen growth [75]. Their use not only aligns with consumer demand for natural ingredients but also supports the industry’s efforts to meet high safety standards in a cost-effective manner [22]. *L. monocytogenes* is not the only concern; there is a substantial list of pathogenic microorganisms causing FBDs each year (Table 3), including many Gram-negative pathogens, such as *E. coli, Campylobacter* sp., and *Salmonella* sp., among others [17].

**Table 3:** Main microorganisms causing foodborne diseases based on the severity of the disease or the number of cases it produces.

Microorganism	Effect	Origin	References
*Campylobacter jejuni*	Most common cause of diarrhea	Raw or undercooked meats and poultry, raw milk, and untreated water.	[57]
*Clostridium botulinum*	Produces botulism, which is characterized by muscle paralysis	Home-prepared foods. Fermented preserved foods.	[6]
*Escherichia coli* O157:H7	Can produce a deadly toxin	Undercooked meats, raw milk, and agricultural products.	[146]
*Listeria monocytogenes*	Causes listeriosis, a severe illness in pregnant women, newborns, and adults with a weak immune system.	Soil and water. It has been found in dairy products, raw and undercooked meat, poultry, and fresh or canned seafood.	[137]
*Salmonella* sp.	It is the leading bacteria causing foodborne illnesses. It is responsible for millions of cases per year of foodborne illnesses.	Raw and undercooked eggs, undercooked poultry and meats, dairy products, seafood, fruits, and vegetables.	[7]
*Staphylococcus aureus*	Produces an enterotoxin that causes vomiting shortly after ingestion	Foods consumed raw, both of animal origin (milk, meat, and eggs) and vegetables (fruits, vegetables, etc.), and ready-to-eat derivative products.	[114]
*Shigella* sp.	Lack of hygiene makes *Shigella* sp. easily transmitted from person to person	Raw foods, especially fruits and vegetables, are more likely to be contaminated with the bacteria if the fields where they are grown are exposed to feces contaminated with the bacteria.	[57]
*Vibrio vulnificus*	Causes gastroenteritis (primary septicemia with liver diseases are at high risk syndrome).	Raw or undercooked oysters.	[141]
*Yersinia enterocolitica*	People causes yersiniosis, a disease characterized by diarrhea and/or vomiting	Raw pork, unpasteurized dairy products, and agricultural products.	[147]

### Use of bacteriocins in the food industry

To prevent foodborne illnesses, various preservation methods have been implemented, each aimed at controlling microbial growth and extending shelf life. These techniques include heat treatments such as pasteurization and sterilization, which effectively reduce or eliminate pathogens, and other approaches involve pH adjustment, reduction in water activity, and the addition of preservatives [83,148]. Common preservatives include antibiotics and organic compounds such as propionate, sorbate, benzoate, lactate, and acetate, which inhibit microbial growth [3]. Together, these methods form a multifaceted approach to food safety, balancing efficacy with the preservation of food quality and sensory attributes [65]. The use of bacteriocins as food preservatives offers several advantages:

1. Extended shelf life: Bacteriocins inhibit the growth of undesirable microorganisms, helping to maintain the freshness and quality of food products over a longer period [18].
2. Additional protection under temperature abuse and other critical control points: Bacteriocins act as an extra barrier, keeping food safe even when storage or transport conditions are not optimal [149].
3. Reduced risk of pathogen spreading through the food chain: By inhibiting pathogenic bacteria, bacteriocins help decrease the likelihood of microbial contamination spreading, thus enhancing safety throughout the food supply chain [150].
4. Decreased economic losses: The use of bacteriocins can reduce food spoilage and the likelihood of recalls or outbreaks related to contaminated products, thus minimizing economic losses for industry [75].
5. Allows for milder processing treatments without compromising safety: Thanks to their antimicrobial capability, bacteriocins enable less intensive processing methods, which better preserve the nutrients, vitamins, and sensory qualities of foods, ultimately improving the product’s quality [10].

These advantages make bacteriocins a natural and effective solution for food preservation. There are at least three methods by which bacteriocins can be integrated into food to improve its safety: adding purified or semi-purified bacteriocin as a food preservative; incorporating an ingredient previously fermented with a bacteriocin-producing strain (starter culture); and inoculating food with the bacteriocin-producing strain [75,76,131].

Incorporating a bacteriocin-producing strain has the disadvantage of lack of compatibility between the strain’s bacteriocin and other cultures needed for fermentation [151]. The use of purified bacteriocins is not always appealing to the food industry, as they must be labeled as additives and are subject to regulatory approval, this labeling requirement can deter manufacturers due to consumer preferences for ‘additive-free’ or ‘clean-label’ products [152]. Additionally, the regulatory approval process can be complex and costly, potentially limiting the widespread adoption of purified bacteriocins as food preservatives [153]. The other two alternatives do not require regulatory approval or labeling as preservatives, making them more appealing to manufacturers, this flexibility supports the industry’s efforts to meet consumer demand for ‘clean-label’ products without the complexities of regulatory compliance, facilitating easier integration into food processing [10]. These options are often considered the most attractive routes for incorporating bacteriocins into food [17]. Bacteriocins can also be incorporated into food packaging films to inhibit spoilage or the growth of pathogenic microorganisms during the storage period of food products [55].

Despite these successes, it is important to recognize that the benefits of adding specific bacteriocins to food products may be constrained by their often-narrow activity spectrum and/or hydrophobic nature [154]. A limited activity spectrum may reduce the effectiveness of certain bacteriocins against a broad range of spoilage organisms or pathogens, while their hydrophobic nature can affect their stability and distribution in food matrices, potentially limiting their overall preservative efficacy [155]. Furthermore, poor solubility or uneven distribution of bacteriocin molecules within food products can significantly affect their antimicrobial efficacy; if bacteriocins do not dissolve well or distribute uniformly throughout the product, their ability to inhibit microbial growth may be compromised, resulting in inconsistent protection against spoilage organisms and pathogens [156]. This limitation can reduce the reliability of bacteriocins as preservatives, particularly in complex food matrices where uniform dispersal is critical for effective preservation. To overcome deficiencies, the use of bacteriocins can be combined with other preservation approaches to enhance their antibacterial activity [111,157]. The FDA regulates the use of bacteriocins and bacteriocinogenic strains under the Federal Food, Drug, and Cosmetic Act, where they are regulated as food ingredients [57].

### Most representative bacteriocins

Currently, nisin is the only bacteriocin approved by the FDA for use as preservatives and anti-spoilage agents in foods [158]. These products are commercially available in the United States and Canada. However, other bacteriocins, such as lacticin and pediocin, show considerable potential as biopreservative agents in the food industry. These bacteriocins have demonstrated broad-spectrum antimicrobial activity against a range of foodborne pathogens and spoilage organisms, enhancing their applicability across various food products [159]. Their stability under different processing conditions also supports their effectiveness as natural preservatives, making them promising candidates for the industry’s shift toward safer, more natural preservation methods (Table 4) [71].

**Table 4:** Bacteriocins as food preservatives.

Bacteriocin	Microorganism	Uses	Doses	Reference
Nisin	*Lactococcus lactis*	Vegetables, drinks, canned food, bakery, fishery, diary, and meat products	25 mg/kg/l	[160]
Pediocin PA-1	*Pedicoccus* sp.	Vegetables, bakery, fishery, diary, and meat products	0.1–1 µg/ml	[161]
Lacticin	*Lactococcus lactis*	Diary and meat products	ND	[162]

ND, no data.

### Nisin

Nisin, first described in 1928, holds historical significance as it was the first bacteriocin isolated from *Lactococcus lactis* subsp. *lactis* [163]. Nisin is the most extensively studied bacteriocin and is widely used as a food preservative due to its strong antimicrobial properties. It holds a unique position as the only bacteriocin recognized by the FDA with GRAS status, underscoring its safety for human consumption [164]. In addition, nisin is approved as a food additive in many countries and is designated with the additive code E234 in the European Union, making it a reliable, regulated option for enhancing food safety and shelf life; the numbering in the International Numbering System for Food Additives is presented in four columns indicating the additive’s identification number, name, functional class, and technological function [165]. It is naturally produced in some dairy products and is used in food production and as an additive in dairy products to prevent decay caused by Gram-positive bacteria, especially from the genera *Clostridium, Staphylococcus, Bacillus*, and *Listeria* [65]. The Codex FAO/WHO Committee on Milk and Dairy Products has authorized the application of nisin as a food additive in processed cheeses, with a limit of 12.5 mg (calculated for pure nisin) per kilogram of product [157]. Nisin is a peptide composed of 34 amino acids, characterized by its low molecular weight of less than 5 kDa. Its small size contributes to its ability to penetrate bacterial cell membranes effectively, allowing it to exert its antimicrobial action [18]. The compact structure of nisin not only enhances its bioactivity but also facilitates its incorporation into various food products, making it a versatile and effective preservative in the food industry [166]. Nisin is classified as a class I lanbiotic, a category of bacteriocins characterized by their peptide structure and specific modes of action, and its composition includes some uncommon amino acids, such as lanthionine, β-methyl-lanthionine, and dihydroalanine, which are not typically found in standard proteins [167]. These unique amino acids contribute to the stability and antimicrobial activity of nisin, enhancing its effectiveness against a broad spectrum of Gram-positive bacteria [168]. The presence of these atypical residues is a key factor in nisin’s functionality, setting it apart from other peptides and making it a valuable tool in food preservation [169]. Nisin is inherently acidic, which contributes to its stability in acidic environments commonly found in many food products [170]. This acidity allows nisin to retain its antimicrobial efficacy in conditions where other preservatives may degrade [18]. Additionally, its solubility increases with rising temperatures and decreasing pH, which enhances its bioavailability and effectiveness as a preservative [171]. This property is particularly advantageous in food processing, where both temperature and pH can be manipulated to optimize the performance of nisin, ensuring that it remains active against pathogenic and spoilage microorganisms [68]. Purified nisin demonstrates remarkable thermal stability, remaining active even after exposure to temperatures of 100°C for 10 minutes at pH 2 [149]. This resilience under high heat and acidic conditions is crucial for its application in food preservation, as it can withstand various processing methods without losing efficacy [146].

The synthesis of nisin is complex, requiring transcription, translation, post-translational modifications, secretion, processing, and transduction signals [172,173]. There are two variants of this bacteriocin, nisin A and nisin Z, which differ only in the amino acid at position 27, histidine in nisin A changes to asparagine in nisin Z [174]. It has been shown that nisin is rapidly inactivated in the intestine by digestive enzymes, which degrade the peptide before it can exert any systemic effects [175]. This rapid inactivation is a significant aspect of nisin’s profile as a food preservative, as it minimizes the risk of any potential adverse effects in humans [176]. Furthermore, studies have indicated that nisin cannot be detected in human saliva just 10 minutes after consuming a liquid containing it, suggesting that it is quickly broken down in the digestive tract [177]. This characteristic not only supports nisin’s safety for consumption but also reinforces its role as a localized antimicrobial agent that functions primarily within food products rather than having a lasting presence in the human body [178].

### Pediocin PA-1

Pediocin PA-1 is a bacteriocin produced by *Pediococcus acidilactici* and is the most representative of the IIa subclass of bacteriocins [179]. It is used as a preservative in vegetable and meat products and has been shown to have high activity against *Listeria* species [180]. The tertiary structure of pediocin PA-1 contains three β-sheets that form a loop conformation and is amphipathic, with positive charge at one end and negative charge at the other [89]. The production of Pediocin PA-1 is limited using expensive media and the low production of native producer strains. Christmann et al. in 2023 [180] conducted a study describing a highly efficient production process of pediocin PA-1 using a genetically modified *C. glutamicum* strain. The production process for pediocin PA-1 has been optimized to achieve high efficiency and high purity, making it a viable option for food preservation applications [181]. Advanced fermentation techniques, such as controlled batch fermentation and optimized growth conditions, contribute to the enhanced yield of *Pediococcus acidilactici*, the bacteriocin-producing strain [182]. Additionally, subsequent purification methods, such as chromatography and filtration, ensure that the final product contains minimal impurities, allowing for concentrated and effective bacteriocin [183]. This high-purity production is crucial for its application in food systems, as it maximizes antimicrobial activity while minimizing the risk of unwanted interactions with other food components, thus enhancing the safety and quality of food products [184].

### Lacticin

Lacticin 3147 is a bacteriocin composed of two peptide chains produced by *Lactococcus lactis*, which is active against a wide variety of Gram-positive bacteria with clinical and food industry relevance [185]. Its two peptides Ltnα and Ltnβ act synergistically by inhibiting peptidoglycan synthesis by forming pores in the bacterial membrane. It is classified as a lantibiotic due to the presence of lanthionine rings within the structure [186]. Ltnα exhibits low bacteriostatic activity when tested alone, with a reported minimum inhibitory concentration required to inhibit the growth of 50% of bacteria (MIC 50) of 200 nM against *L. lactis* HP [187]. However, when combined in a 1:1 molar ratio with Ltnβ, the MIC50 falls to 7 nM, indicating optimal joint activity [188]. The two peptides of lacticin 3147 act in a 1:1 ratio; Ltnα binds to the precursor for peptidoglycan production, lipid II, with Ltnβ subsequently interacting with the complex, thus forming a depolarizing pore in the membrane [189]. Recently, it has been confirmed that lacticin 3147 can inhibit *L. monocytogenes* in set-style soft cheeses and control the growth of non-starter LAB in Cheddar cheese [186].

Further research using this whey powder containing lacticin 3147 revealed that its presence resulted in an 85% and 99.9% reduction in *L. monocytogenes* populations in cottage cheese and plain yogurt, respectively, within a 2-hour period, *B. cereus* numbers were also observed to be reduced by 80% within a 3-hour period in a soup containing this powder [190]. Regarding the heat sensitivity of lacticin 3147 powder, similarly to the situation with Nisaplin®, a compensation factor could be developed to account for the loss of activity upon heat treatment [191].

### Drawbacks of using bacteriocins

Despite the use of bacteriocins as a preservative in food is well known, they present three principal drawbacks: 1. Bacteriocins have a narrow action spectrum related to the foodborne pathogens in comparison with current preservatives [192]. 2. The feasibility of large-scale production of some bacteriocins as class IIa is low because the amount of bacteriocin produced by a cell is not enough for the necessities in the food industry; besides the cost is high in the production and purification of bacteriocins compared with the traditional methods [193]. 3. Due to their proteic nature, some bacteriocins are susceptible to proteolytic activity and high temperatures [194].

### Regulatory framework

**Figure 2:**
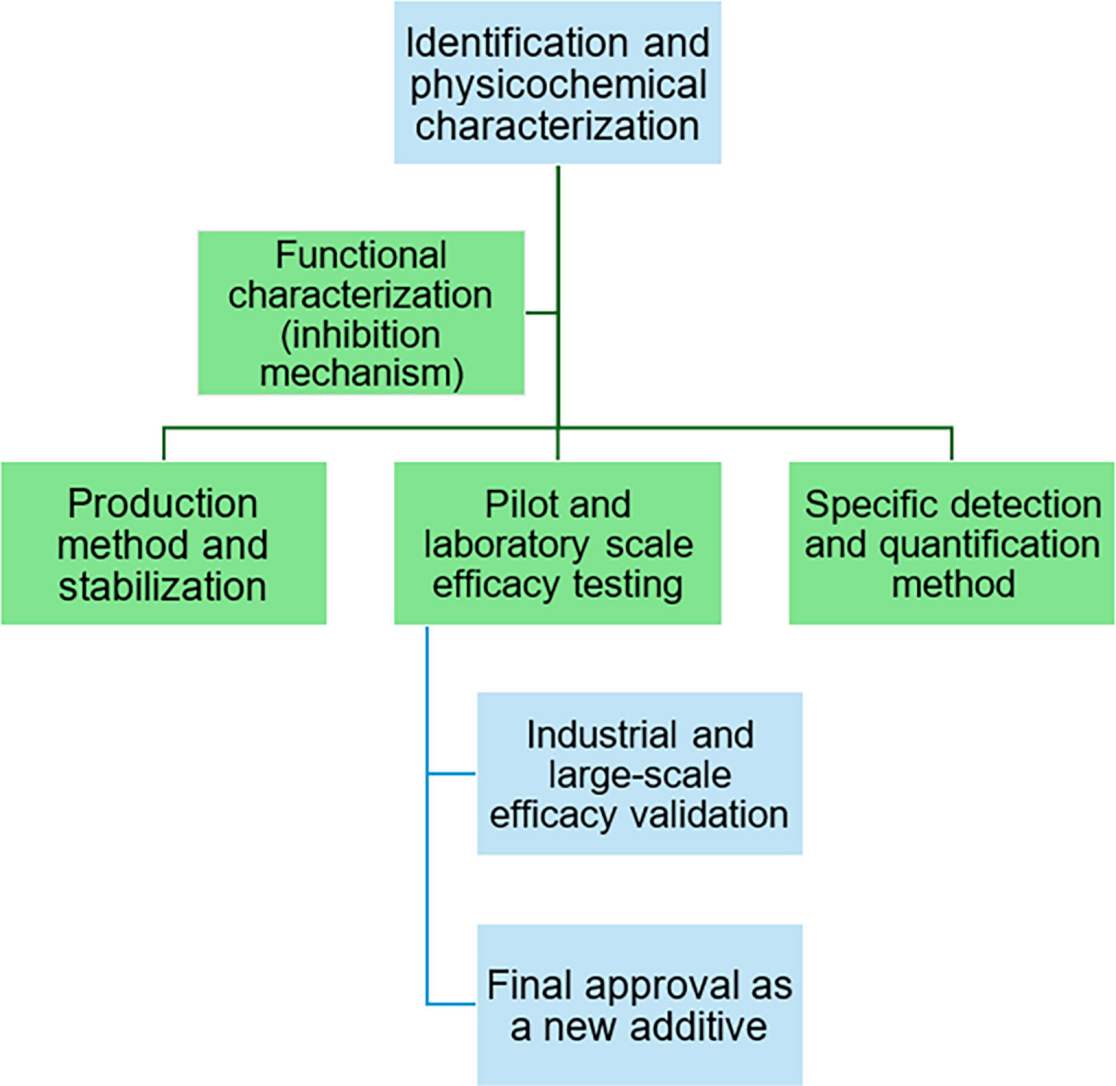
Guide for the evaluation and approval of new bacteriocins for food applications. A diagram is presented illustrating the essential steps for evaluating and approving new bacteriocins intended for use in food applications.

Title 21 of the Code of Federal Regulations (CFR) establishes regulations for food additives, including bacteriocins [195]. According to the CFR, any company using purified bacteriocin as a food preservative must be declared GRAS by the FDA. The FDA requires justification for this self-proclamation [142]. The European Union assigns ‘E numbers’ to all food additives, including bacteriocins [196]. To be approved as an additive, a new bacteriocin must undergo several evaluation steps. The approval application for a new bacteriocin must include the following criteria (Figure 2) [10]:

The new bacteriocin must have its identity and chemical composition determined, implying high purity of the active molecule and the amino acid sequence established using standard techniques of biochemistry and molecular biology [117,197]. The method used to prepare and stabilize it must be specified [198]. Likewise, a statement including the recommended concentration for its use and the intended purpose, along with all relevant instructions and recommendations, is required [183]. An appropriate analysis method must be employed to determine the final concentration in the finished food, as well as to provide data demonstrating its intended efficacy [198]. Furthermore, comprehensive reports on the safety of the bacteriocin under recommended conditions of use are needed, including assessments of acute and subacute toxicity, as well as potential long-term effects [199]. Data on the acceptable residual concentration in the final product must be provided, and a proposed maximum concentration for the additive or bacteriocin in the finished food product must be established [10,75].

The exploration of new food preservatives leads us to the applications of bacteriocin-producing bacteria as an innovative model; since they are cationic proteins secreted by bacteria that are capable of inhibiting the growth of pathogens that can cause some FBDs, the target of these cationic proteins mainly form pores in the cell membrane of the target bacteria or inhibit protein synthesis and DNA replication [81,88]. Bacteriocins derived from LAB have GRAS status; for example, nisin promises safe use as a food preservative in vegetables, dairy products, cheeses, meats, and other food products, since it inhibits contamination by microorganisms in the production process [143].

## Conclusions and perspectives

Bacteriocins represent a promising strategy for food preservation, offering a natural and effective alternative against foodborne pathogens. Although significant progress has been made in their characterization, mechanisms of action, and applications in different food matrices, challenges related to large-scale production, purification, and compliance with international regulations limit their widespread implementation. In the future, efforts should focus on developing more efficient and sustainable technologies for their production, combining bacteriocins with other preservation methods to enhance their effectiveness, and conducting long-term studies to support their safety and impact on the sensory properties of food. Furthermore, regulatory approval in various regions worldwide will be crucial to expand their commercial use and establish them as key tools for food preservation in a globalized market demanding safe, natural, and sustainable solutions.

## Abbreviations

AOIs: areas of interest
FBDs: foodborne diseases
GRAS: generally regarded as safe
LAB: lactic acid bacteria
ORFs: Open Reading Frames
RiPPs: ribosomally synthesized and post-translationally modified peptides
